# A chloroplast genomic dataset for accurate identification of the endangered *Lagerstroemia minuticarpa* Debb. ex P. C. Kanjilal

**DOI:** 10.1016/j.dib.2026.112724

**Published:** 2026-03-26

**Authors:** Kai Mu, Jinling Wang, Chao Xu, Jin Zhang, Xueying Yang, Ruijian Wang

**Affiliations:** a Engineering Research Centre of Forestry Biotechnology of Jilin Province, College of Forestry, Beihua University, Jilin 132013, China; b Key Laboratory of Systematic and Evolutionary Botany/ State Key Laboratory of Plant Diversity and Specialty Crops, Institute of Botany, Chinese Academy of Sciences, Beijing 100093, China; c China National Botanical Garden, Beijing 100093, China; d National Engineering Laboratory for Forensic Science, Key Laboratory of Forensic Genetics, Institute of Forensic Science, Ministry of Public Security, Beijing 100038, China

**Keywords:** **Lagerstroemia minuticarpa**, Chloroplast genome, Phylogenetic analysis, Lythraceae, Molecular marker

## Abstract

*Lagerstroemia minuticarpa* Debb. ex P. C. Kanjilal is a deciduous tree listed as a nationally protected wild plant (level II) in China. The complete chloroplast genome of L. *minuticarpa* was sequenced using the DNBSEQ-T7 platform. The genome is 152,183 bp in length and exhibits a typical quadripartite structure, consisting of a large single-copy (LSC) region of 84,009 bp, a small single-copy (SSC) region of 16,924 bp, and a pair of inverted repeats (IRs) of 25,625 bp each. It contains 133 genes in total, including 85 protein-coding genes, 37 transfer RNA genes, and 8 ribosomal RNA genes. The overall GC content of the genome is 37.31%. Phylogenetic analysis based on 39 chloroplast genomes revealed that *L. minuticarpa* forms an independent lineage nested within the genus *Lagerstroemia*. Additionally, pairwise alignment identified ten highly variable chloroplast loci (including both coding and intergenic regions) with elevated parsimony-informative sites, which hold promise as specific DNA barcodes for species identification in *Lagerstroemia*. These data enrich the genetic knowledge of *Lagerstroemia* species in the Lythraceae family and provide a basis for further research in molecular identification, genetic breeding, and other related fields.

Specifications Table

**Table:** 

Subject	Biology
Specific subject area	Omics: Chloroplast Genomics.
Type of data	Tables, Figures. Raw, Analyzed.
Data collection	Dried leaf materials were sampled from a herbarium specimen, and total genomic DNA was extracted using a modified CTAB method. Whole genome sequencing was performed on a DNASEQ-T7 sequencer. Chloroplast genome was assembled using GetOrganelle v1.7.7.1. Annotation was performed using Plann v1.1. A circular genome map was generated using the OGDRAW program. Multiple sequence alignment was conducted using MAFFT v7.055b A maximum likelihood phylogenetic tree was constructed with IQ-TREE v2.3.6 under the optimal nucleotide substitution model selected by ModelFinder Plus. The number of conservative, variable, and parsimony informative sites were calculated in MEGA v12. The value of Pi was inferred in DnaSP v6.12.03.
Data source location	City: Beijing City. Country: China. Latitude and longitude: 116.21616, 39.99192. Voucher Specimen: Deposited at Herbarium of Institute of Botany, Chinese Academy of Sciences under voucher number PE-01,129,215.
Data accessibility	Repository name: NCBI. The NCBI links to the sequenced data can be accessed at: https://www.ncbi.nlm.nih.gov/nucleotide/PZ011249 https://www.ncbi.nlm.nih.gov/sra/PRJNA1424257 https://www.ncbi.nlm.nih.gov/biosample/?term=SAMN55380968 https://www.ncbi.nlm.nih.gov/sra/?term=SRR37298158 Repository name: Science Data Bank. The Science Data Bank links to the analyzed data can be accessed at: https://doi.org/10.57760/sciencedb.28565
Related research article	None.

## 1 Value of the Data

• The chloroplast genome data provides foundational genomic resources for assessing germplasm, investigating population genetics, and informing conservation strategies for *Lagerstroemia minuticarpa*.
• The complete chloroplast genome and newly identified highly variable loci enables accurate species identification and robust phylogenetic inference within *Lagerstroemia*.
• These genomic data will facilitate broader advances in molecular botany, genomics, and bioinformatics, serving as a key dataset for systematic and evolutionary studies in the genus.

## 2 Background

**Fig. 1:**
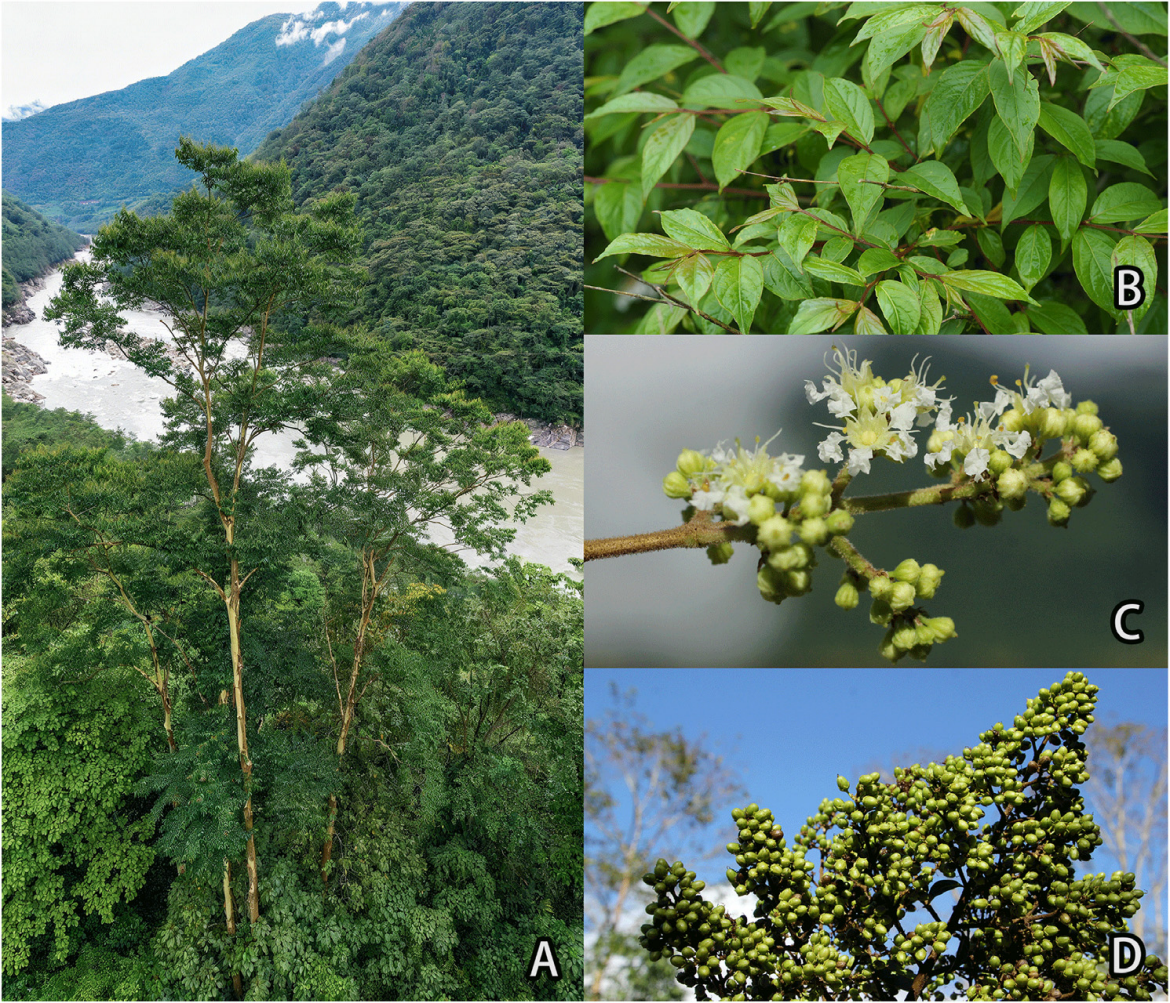
Morphology and habitat of the *Lagerstroemia minuticarpa*. (A) Habitat along a river valley (photo by Zi Wang). (B) Close-up of leaves (photo by Yechun Xu). (C) Inflorescence with open flowers and developing buds (photo by Xirong Zheng). (D) Mature fruit clusters (photo by Xirong Zheng).

*Lagerstroemia minuticarpa* Debb. ex P.C. Kanjilal is a threatened deciduous tree species belonging to the family Lythraceae (Fig. 1) . The species exhibits a disjunct distribution, being endemic to restricted areas in southwestern China and northeastern India. Within China, its natural populations are confined to Motuo County in the Xizang Autonomous Region and Gongshan County in Yunnan Province. Due to its extremely narrow distribution range, sparse wild populations, and evidence of continuous population decline, *L. minuticarpa* has been listed as a national second-class protected plant under the List of National Key Protected Wild Plants in China. According to the IUCN Red List Criteria (ver. 3. 1), it is classified as Critically Endangered under criterion C2a (i) +2b [1], reflecting an estimated population size of fewer than 250 mature individuals and a continuing decline. This critical status necessitates urgent conservation interventions and legal protection at the national level. Beyond its conservation significance, *L. minuticarpa* possesses considerable ornamental value, characterized by its graceful architecture and attractive flowers, which render it a potential candidate for horticultural applications [2].

Chloroplast genomes are widely used in plant systematic research due to their maternal inheritance, haploid nature, highly conserved structure, and slower evolutionary rate [3]. Although complete chloroplast genome sequences have been reported for several congeneric species, including the ornamental staples *Lagerstroemia indica* and *Lagerstroemia loudonii* [4,5], no genomic data for *L. minuticarpa* are currently available in public repositories such as the NCBI GenBank database. This knowledge gap impedes molecular evolutionary studies essential for monitoring this critically endangered species. The generation of genome data for *L. minuticarpa* is therefore urgently needed to document its valuable genetic resources. Furthermore, comparative genomic analyses across *Lagerstroemia* species will elucidate patterns of sequence divergence, clarify phylogenetic relationships among closely related taxa, and enable the development of robust molecular markers for accurate species identification within this taxonomically challenging genus.

## 3 Data Description

Whole genome sequencing produced approximately 16.3 GB of raw data, consisting of 26,027,219 paired-end reads with a GC content of 52.8%. The overall sequencing quality was high, with Q20 and Q30 scores of 99.2% and 98.1%, respectively.

**Fig. 2:**
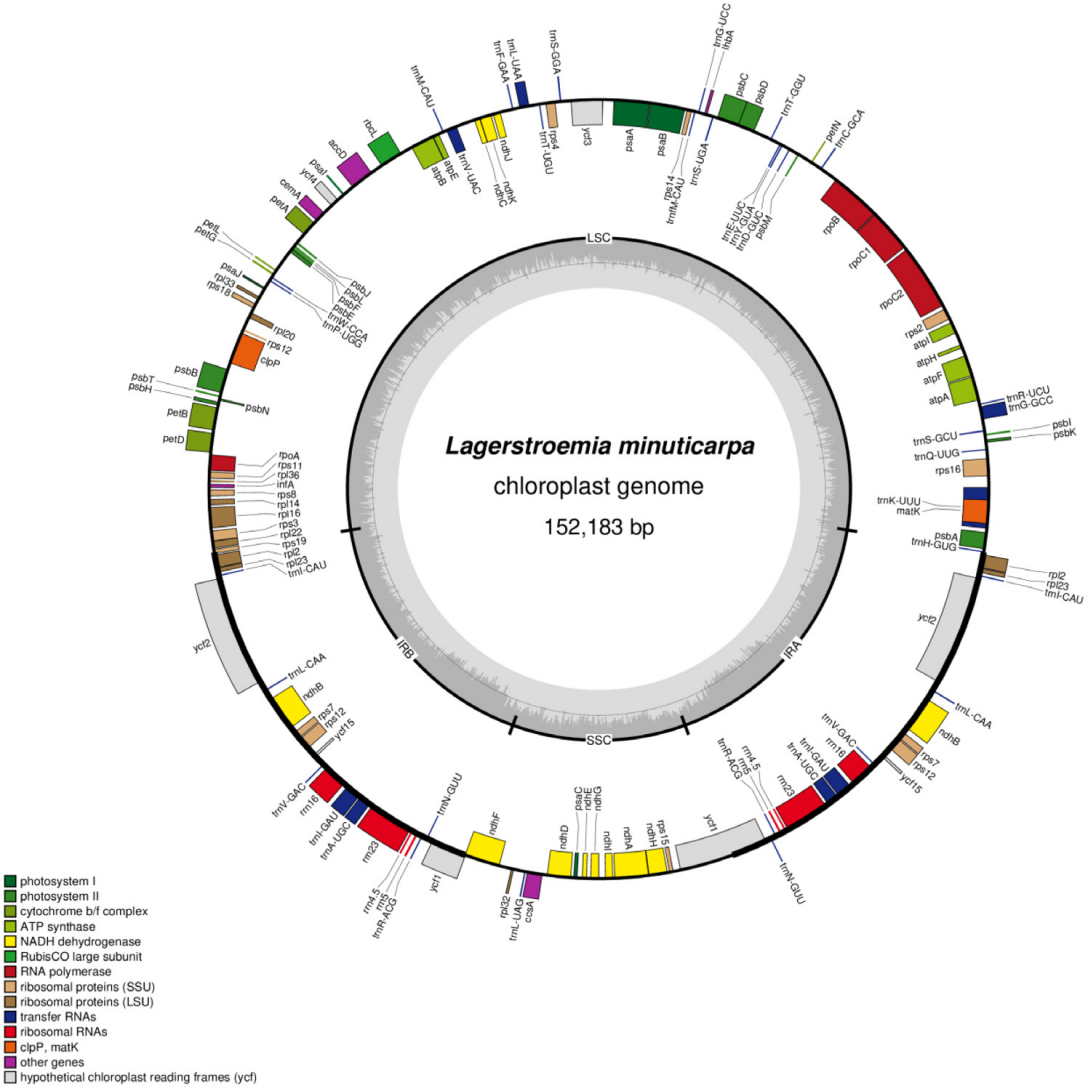
Circular map of *Lagerstroemia minuticarpa* chloroplast genome. Genes are color-coded by function. Genes located on the outside are transcribed counter-clockwise, while those on the inside are transcribed clockwise. The dashed area within the grey inner circle represents the GC content across the genome, with the inner circle line indicating the 50% GC content threshold.

The total length of the chloroplast genome of *Lagerstroemia minuticarpa* was 152,183 bp, with a GC content of 37.31 %. The chloroplast genome was divided into four main regions: LSC (large single-copy region), length of 84,009 bp. SSC (small single-copy region), length of 16,924 bp (Fig. 2). IRa and IRb (reverse repeat regions), each region was 25,625 bp in length. It contains 133 genes (Table 1), including 85 protein-coding genes, 37 tRNA genes and eight rRNA genes.

**Table 1:** Classification of the *Lagerstroemia minuticarpa* genes after annotation of the chloroplast genome. The annotated genes were categorized according to their function.

Category	Gene group	Gene name
Photosynthesis	Subunits of photosystem I	*psaA, psaB, psaC, psaI, psaJ*
	Subunits of photosystem II	*psbA, psbB, psbC, psbD, psbE, psbF, psbH, psbI, psbJ, psbK, psbL, psbM, psbN, psbT, lhbA* (*psbZ*)
	Subunits of NADH dehydrogenase	ndhA*, ndhB* (2), *ndhC, ndhD, ndhE, ndhF, ndhG, ndhH, ndhI, ndhJ, ndhK*
	Subunits of cytochrome b/f complex	petA, petB*, petD*, petG, petL, petN
	Subunits of ATP synthase	atpA, atpB, atpE, atpF*, atpH, atpI
	Large subunit of rubisco	*rbcL*
Self-replication	Proteins of large ribosomal subunit	rpl2* (2), rpl14, rpl16*, rpl20, rpl22, rpl23 (2), *rpl32, rpl33, rpl36*
	Proteins of small ribosomal subunit	*rps2, rps3, rps4, rps7* (2), rps8, rps11, rps12** (2), rps14, rps15, rps16*, rps18, rps19
	Subunits of RNA polymerase	rpoA, rpoB, rpoC1*, rpoC2
	Ribosomal RNAs	*rrn4. 5* (2), *rrn5* (2), *rrn16* (2), *rrn23* (2)
	Transfer RNAs	trnA-UGC* (2), trnC-GCA, trnD-GUC, trnE-UUC, trnF-GAA, trnG-GCC*, trnG-UCC, trnH-GUG, trnI-CAU (2), trnI-GAU* (2), trnK-UUU*, trnL-CAA (2), trnL-UAA*, trnL-UAG, trnM-CAU, trnN-GUU (2), *trnP-UGG, trnQ-UUG, trnR-ACG* (2), *trnR-UCU, trnS-GCU, trnS-GGA, trnS-UGA, trnT-GGU, trnT-UGU, trnV-GAC* (2), trnV-UAC*, trnW-CCA, trnY-GUA, trnfM-CAU
Other genes	Maturase	*matK*
	Protease	clpP**
	Envelope membrane protein	*cemA*
	Acetyl-CoA carboxylase	accD*
	c-type cytochrome synthesis gene	*ccsA*
	Translation initiation factor	#*infA*
Genes of unknown function	Conserved hypothetical chloroplast ORF	*ycf1* (2), *ycf2* (2), ycf3**, ycf4, #ycf15 (2)
*Notes:* Gene*: Gene with one introns; Gene^⁎⁎^: Gene with two introns; #Gene: Pseudo gene; Gene (2): multi-copy genes (copy number).		

**Fig. 3:**
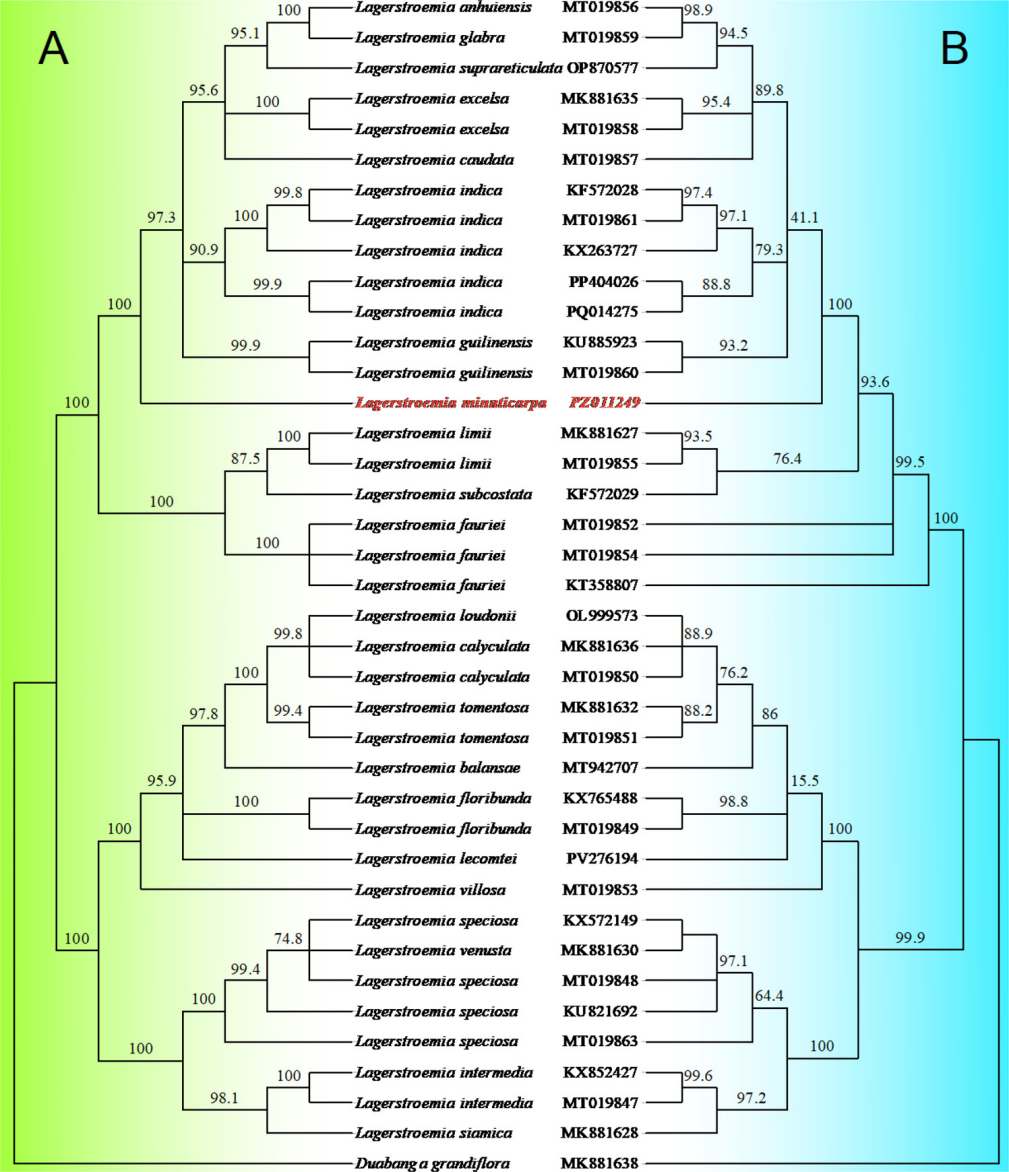
The phylogenetic trees, based on the maximum-likelihood method, with *Duabanga grandiflora* as the outgroup. The left tree in green (A) was constructed using the complete chloroplast genome sequences of *Lagerstroemia* species. The right tree in blue (B) was constructed using sequence matrix concatenated from ten highly variable genes and intergenic regions in the *Lagerstroemia* chloroplast genomes. Numbers next to the nodes represent bootstrap support values. The endangered *L. minuticarpa* was colored in red.

To elucidate the evolutionary connection between *Lagerstroemia minuticarpa* and other congeneric species, a phylogenetic tree was constructed based on complete chloroplast genome sequences, with *Duabanga grandiflora* as the outgroup (Fig. 3A). The phylogenetic tree exhibited clear branching topology, with most species clades supported by high bootstrap values. *Lagerstroemia minuticarpa* (PZ011249) formed a distinct independent branch with 100 % bootstrap support, without clustering with any other species in the genus, while it showed a relatively close evolutionary affinity to other *Lagerstroemia* species endemic to southwestern China.

Ten loci with the highest parsimony-informative site numbers were selected from 213 chloroplast genes and intergenic regions of 38 *Lagerstroemia* species (Table S1), aligned, and concatenated into a 15,419 bp sequence matrix for phylogenetic analysis. The tree inferred from these concatenated sequences showed consistent overall interspecific phylogenetic relationships with that of the complete chloroplast genome, with a slight reduction in branching resolution (Fig. 3).

## 4 Experimental Design, Materials and Methods

### 4.1 Plant materials and DNA extraction

Specimen of *Lagerstroemia minuticarpa* was collected from an evergreen broad-leaved forest on a hillside in Dulongjiang Township, Gongshan County, Yunnan Province, China, in 1982. The voucher specimen (PE-01,129,215) was deposited at the herbarium of Institute of Botany, Chinese Academy of Sciences. Total genomic DNA was extracted from a single mature leaf tissue from the specimen using a modified CTAB method [6]. DNA quality and concentration were assessed by electrophoresis on a 1% (w/v) agarose gel. As the DNA originates from a single individual, it represents only one genotype and may not capture the full intraspecific diversity of *Lagerstroemia minuticarpa*.

### 4.2 Sequencing and sequence analyses

The genome sequencing was conducted on the DNBSEQ-T7 platform (BGI Genomics, China) following the manufacture’s protocol. The raw sequence data quality was evaluated by FastQC v0.12. 1 [7] package with the default parameters. The filtered reads were then assembled into a circular chloroplast genome using GetOrganelle v1.7.7.1 [8]. Plann v1.1 [9] was used to annotate the assembled genome using *Lagerstroemia guilinensis* (NCBI accession number: MT019860) as reference to predict protein-coding genes as well as tRNA and rRNA genes. A circular genome map was generated using OGDRAW v1.3.1 [10]. The complete chloroplast genome of *L. minuticarpa* has been deposited in GenBank under the accession number PZ011249. The raw sequencing fastq files were uploaded to the NCBI SRA database. The associated BioProject, SRA, and BioSample numbers are PRJNA1424257, SRR37298158, and SAMN55380968, respectively.

### 4.3 Phylogenetic analysis

In this study, a phylogenomic analysis was performed using the complete chloroplast genome sequences of *L. minuticarpa* and 22 other *Lagerstroemia* species, with *Duabanga grandiflora* as an outgroup (Fig. 3A). The GenBank accession numbers of all sequences used are included in Fig. 3. Multiple sequence alignment was conducted using MAFFT v7.055b [11]. A maximum likelihood (ML) phylogenetic tree was constructed with IQ-TREE 2.3.6 [12] under the optimal nucleotide substitution model selected by ModelFinder Plus (MFP). Node support was evaluated using 1000 ultrafast bootstrap replicates with the Boost NNI (–bnni) correction and 1000 SH-aLRT tests. The aligned dataset of whole chloroplast genomes was deposited at https://doi.org/10.57760/sciencedb.28565.

### 4.4 Comparative chloroplast genome analysis

Genes and intergenic regions of the 38 *Lagerstroemia* chloroplast genomes were extracted according to the annotated information. Sequences that were too short and were not included in comparison. We got 213 gene and intergenic region datasets in total (Table S1). All the datasets were aligned using MAFFT v7.055b before analysis. Constant Site, Variable site, Parsimony-informative site, Singleton Sites in 213 datasets were identified in MEGA (12.1.2), and their nucleotide diversity (Pi, π) was calculated with DnaSP (6.12.03). Ten highly variable genes and intergenic regions, which had the highest parsimony-informative site numbers, were screened out (Table 2), concatenated, and used to construct a phylogenetic tree following the method described in Section 4.3 (Fig. 3B). The aligned datasets of 213 gene and intergenic region were deposited at https://doi.org/10.57760/sciencedb.28565.

**Table 2:** Ten highly variable genes and intergenic regions screened from the chloroplast genomes of 38 *Lagerstroemia* accessions.

Locus	No. of Sites	No. of NetSites	No. of Constant Sites	No. of Variable Sites	No. of Parsimony-informative Sites	No. of Singleton Sites	Nucleotide Diversity Pi (π) (Nei 1987)
*ndhF*	2244	2244	2179	65	56	9	0. 0096
*trnK*	2571	2556	2511	58	49	9	0. 0076
*trnS*-*trnR*	1610	1569	1557	52	47	5	0. 0122
*rpl32*-*trnL*	716	639	651	49	43	6	0. 0253
*ndhF*-*rpl32*	798	666	734	60	43	17	0. 0225
*rpoC2*	4179	4164	4131	48	41	7	0. 0038
*matK*-*rps16*	1505	1401	1457	46	40	6	0. 0101
*petA*-*psbJ*	638	608	597	41	37	4	0. 0266
*trnI*	1031	914	969	61	36	25	0. 0150
*rrn16*-*trnI*	293	239	259	34	33	1	0. 0463

## Limitations

None.

## Ethics Statement

All authors have read and follow the ethical requirements for publication in Data in Brief and confirming that the current work does not involve human subjects, animal experiments, or any data collected from social media platforms.

## CRediT Author Statement

**Kai Mu:** Conceptualization, Methodology, Software, Original draft preparation; **Jinling Wang:** Visualization, Investigation; **Chao Xu:** Data curation, Writing; **Jin Zhang:** Supervision, Validation; **Xueying Yang:** Funding, Writing- Reviewing and Editing; **Ruijian Wang:** Supervision, Project administration, Writing.

## Data Availability

NCBI BiosamplePlant sample from Lagerstroemia minuticarpa (Original data). GenBankLagerstroemia minuticarpa voucher PE01129215 chloroplast, complete genome (Original data).

## Appendix Supplementary Materials

Image, application 1
